# A Low Viral Dose in COVID-19 Patient: A Case Report

**DOI:** 10.3389/fpubh.2020.00339

**Published:** 2020-06-26

**Authors:** Yajuan Li, Xianwei Hu, Youhui Tu, Tao Wu, Bo Wang, Huan Ma, Weihong Zeng, Dan Zhao, Hylemariam Mihiretie Mengist, Arnaud John Kombe Kombe, Meijuan Zheng, Yuanhong Xu, Tengchuan Jin

**Affiliations:** ^1^Department of Clinical Laboratory, The First Affiliated Hospital of Anhui Medical University, Hefei, China; ^2^Department of Respiratory and Critical Care Medicine, The First Affiliated Hospital of Anhui Medical University, Hefei, China; ^3^Department of Nuclear Medicine, The First Affiliated Hospital of Anhui Medical University, Hefei, China; ^4^Hefei National Laboratory for Physical Sciences at Microscale, Laboratory of Structural Immunology, CAS Key Laboratory of Innate Immunity and Chronic Disease, Division of Life Sciences and Medicine, University of Science and Technology of China, Hefei, China; ^5^Division of Molecular Medicine, Division of Life Sciences and Medicine, Department of Obstetrics and Gynecology, The First Affiliated Hospital of USTC, University of Science and Technology of China, Hefei, China; ^6^CAS Center for Excellence in Molecular Cell Science, Chinese Academy of Science, Shanghai, China

**Keywords:** COVID-19 patient, RT-qPCR, IgA, IgM, IgG

## Abstract

SARS-CoV-2 outbreak has attracted global attention. Verifying the presence of viral RNA is the gold standard for the diagnosis of COVID-19. However, RT-qPCR diagnosis often fails to catch infected patients, because of inconsistent swab sample collection. Here we report a case that showed 5 consecutive negative and 1 low-viral- dose RT-qPCR results during illness spanning over 20 days. Clinical symptoms suggest SARS-CoV-2 infection with typical ground glass like a lung in computed tomography. SARS-CoV-2 infection was serologically confirmed by the presence of anti-SARS-CoV-2 specific antibodies in patients' serum. Finally, a high level of protective IgG was produced after the patient recovered. Surprisingly, as a barber and a housewife staying at home for the first 2 weeks after the onset of illness, none of the close contacts were infected, showing a case of low viral load and low infectivity in this patient.

## Highlights

- A COVID-19 patient with consistently negative RT-qPCR results.
- Lack of SARS-CoV-2 transmission from a patient with COVID-19 clinical symptoms to close contacts.
- Serum antibody detection confirmed SARS-CoV-2 infection.

## Introduction

The Severe acute respiratory syndrome coronavirus-2 (SARS-CoV-2) is thought to be transmitted through respiratory droplets and direct contact with the novel coronavirus disease (COVID-19) patients (1). The COVID-19-related symptoms are not specific and studies reported about 40% of the confirmed cases of SARS-CoV-2 infection were asymptomatic at the early stage of infection (2). The symptomatic patients produced nearly 50% false-negative RT-qPCT for SARS-CoV-2 ribonucleic acid (3), which brings complexity and may delay diagnosis and treatment which in turn may lead to considerably increased spread of infection.

To make a definite diagnosis for SARS-CoV-2 infection, we reported a case of COVID-19 patient with consistently negative or low-doses of SARS-CoV-2 RNA from oropharyngeal swabs and sputum samples during the whole course of illness. Finally, serum antibody detection confirmed SARS-CoV-2 infection, which demonstrates an alternative diagnostic method for COVID-19.

## Case Presentation

On January 27, 2020, a 40-year-old female presented symptoms of “myalgia, chills, and fever (>37.9°C).” On January 30, the patient went to the local health center and was treated with intravenous infusions for 3 days, which did not improve her fever and dry cough after physical activities, and even after taking anti-fever medicines as well.

On February 5, the patient went to the fever clinic of the First Affiliated Hospital of Anhui Medical University for further evaluation. The chest computed tomography (CT) results showed multiple high-density irregular shadows in both lungs. The patient reported not having traveled or having resided in and/or around Wuhan or other communities with reported cases or not having been exposed to other patients with fever or respiratory symptoms during the 14 days before disease onset. She also said not having clustered with people or been in close contact with anyone with a known SARS-CoV-2 infection, and none of her family members had been diagnosed infected with SARS-CoV-2.

According to the travel history and examination results, as well as multidisciplinary consultation, she was not suspected to be a COVID-19 patient and was advised for home quarantine with her family with a follow-up visit. On February 6, her body temperature rose to 38°C accompanied by myalgia and asthenia but without nausea, vomiting, or diarrhea. The patient visited the fever clinic again on February 7.

**Figure 1:**
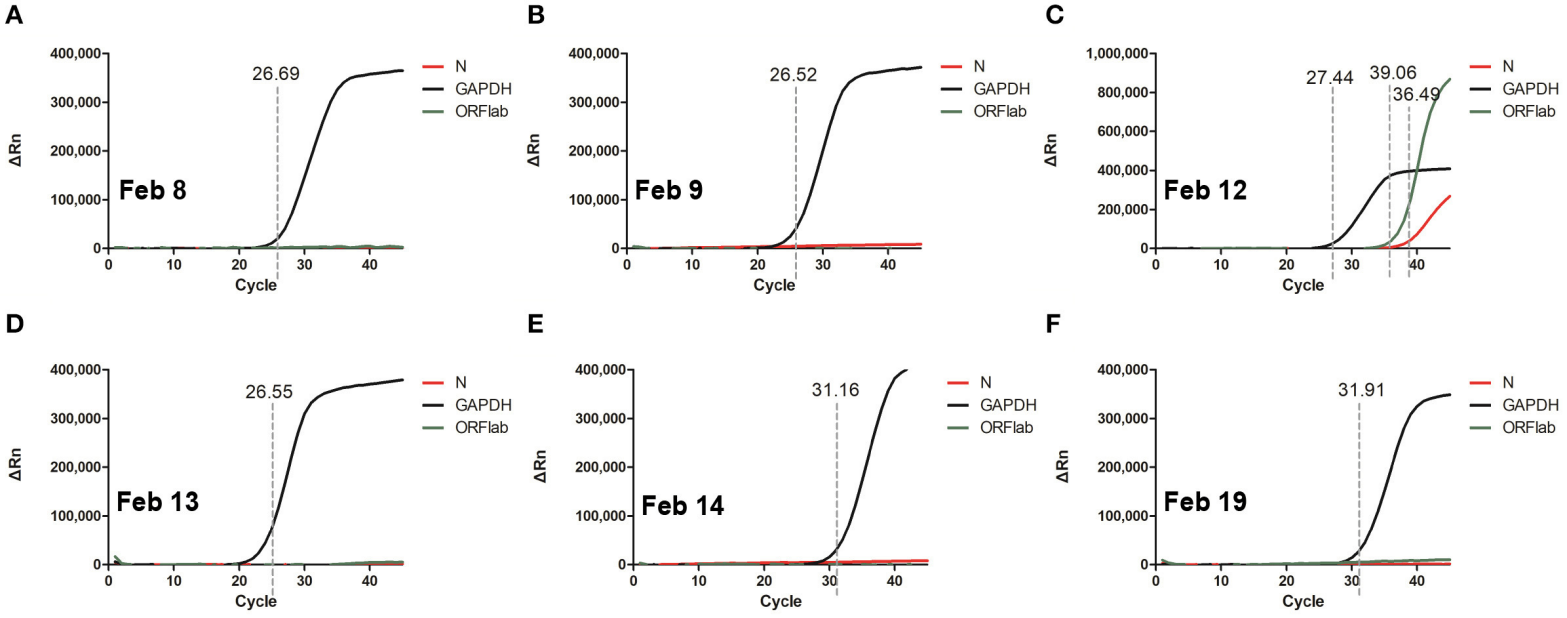
RT-qPCR of SARS-CoV-2 nucleic acid with patient throat swab and sputum specimens on Feb 8 **(A)**, Feb 9 **(B)**, Feb 12 **(C)**, Feb 13 **(D)**, Feb 14 **(E)**, Feb 19 **(F)**. Two target genes of SARS-CoV-2 including open reading frame 1ab (*ORF1ab*) and Nucleocapsid protein (N) marked as FAM and VIC channel, respectively, are simultaneously tested to report a positive gene. Human GAPDH indicates a reference gene. Ct value were demonstrated on the diagram.

**Figure 2:**
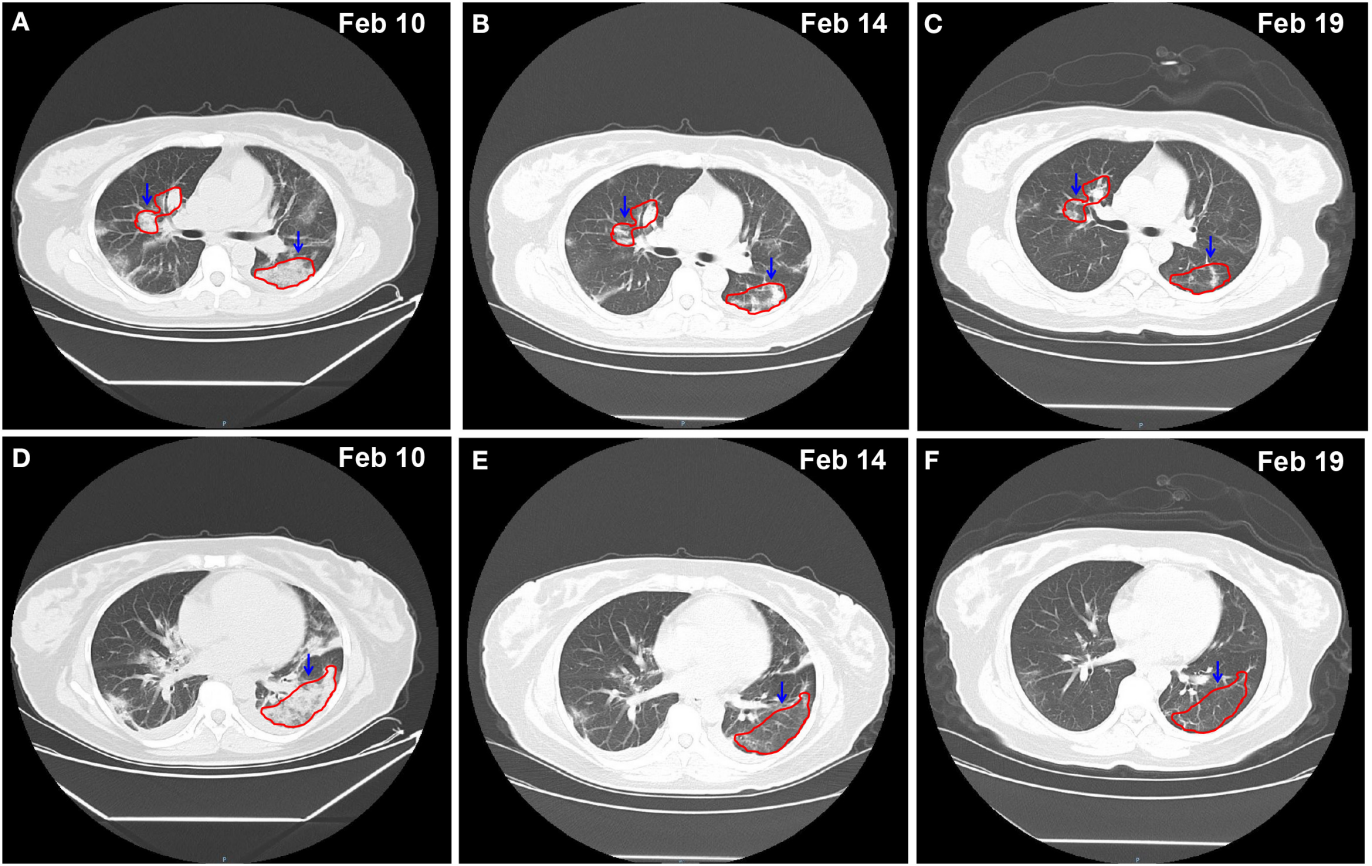
Chest CT imaging of the patient on Feb 10 -Feb 19, 2020. **(A–C)** pulmonary trunk as the reference coordinate, **(D–F)** tracheal bifurcation as the reference coordinate. CT, computed tomography. Red circle indicates the focus of lesion.

In order to improve the detection rate of SARS-CoV-2 nucleic acid, the samples of each patient from nasopharyngeal swabs and sputum were mixed. The two consecutive RT-qPCR tests from mixed samples were both negative on February 8 and 9, respectively (Figures 1A,B). The administered combination of moxifloxacin and oseltamivir did not improve her health state. Chest CT results showed aggravating lesions of bilateral pneumonia on February 10, with a little pleural effusion (Figures 2A,D, red coil).

This patient was admitted to the Department of Respiratory and Critical Care for in-depth treatment. On admission, the patient presented fever, fatigue, myalgia, combined with respiratory failure (type I) with low pO_2_ 57 mmHg and K^+^ 2.8 mM (Table 1). It was highly suspected that the patient was infected by SARS-CoV-2. The clinical examinations including total white cell count, lymphocyte count, D-dimer, etc. were all normal, while the C reactive protein (CRP) was slightly elevated.

**Table 1:** Clinical information of the patient during hospitalization.

**Date**	**Feb 11-12**	**Feb 13-14**	**Feb 19-20**	**Reference range**
Day after onset	16	18	25	
Body temperature	Fever	Fever	Normal	Normal
RBC (×10^12^/L)	4.56	4.28	4.69	3.80–5.10
WBC (×10^9^/L)	4.22	5.43	7.15	3.50–9.50
Neutrophil (×10^9^/L)	2.22	3.24	4.23	1.80–6.30
Neutrophil (%)	52.60	59.7	59.2	40.00–75.00
Lymphocytecount (×10^9^/L)	1.60	1.76	2.41	1.10–3.20
Lymphocyte (%)	37.90	32.4	33.7	20.00–50.00
Hemoglobin (g)/L	107	103	113	115-150
Platelet count (×10^9^/L)	351	380	425	125–350
PT(s)	/	13.3	13.3	11.0–16.0
APTT (s)	/	31.1	31.3	28–42
ALT (U/L)	23	18	15	7–40
AST (U/L)	29	22	15	13–35
Total bilirubin (μM)	6.63	9.3	14.1	0.0–23.0
Potassium (mM)	**2.80**	4.02	4.02	3.50–5.30
Sodium (mM)	137	140	137.5	137.0–147.0
Creatinine (μM)	43.2	45	45	41.0–73.0
BUN (mM)	3.58	3.10	4.92	2.60–7.50
Blood glucose (mM)	5.18	4.49	4.1	3.89–6.11
Procalcitonin (ng/L)	<0.05	<0.05	<0.05	0.00–0.50
C reactive protein (mg/L)	**67.83**	**24.1**	5.9	0.00–3.00
Creatinekinase (U/L)	33	31	17	40–200
CKMB (U/L)	7	6	7	0–24
IL-6 (pg/ml)	<2.000	**20.1**	8.49	0.000–5.900
D-Dimer	/	2.54	1.12	0.00–0.50
Myo(ng/ml)	7	9	/	10.00–46.00
cTn (ng/ml)	<0.010	<0.010	/	0.000–0.034
Lac (mmol/L)	1.0	1.3	2.9	1.0–1.8
pO_2_ (mmHg)	**57**	64.2	188.2	83–108
pCO_2_ (mmHg)	37	34.7	33.5	32.00–48.00

*WBC, white blood cell count; PT, prothrombin time; APTT, activated partial thromboplastin time; ALT, alanine aminotransferase; AST, aspartate aminotransferase; BUN, blood urea nitrate; CKMB, creatine kinase-MB; IL-6, interleukin-6. The bold values indicated special abnormal values*.

Then, On February 12, sputum obtained by induction with inhalation of atomized hypertonic saline was tested with RT-qPCR. The result was weak positive; however, the viral load was low with Ct values of 39.05 and 36.45, for Nucleocapsid protein (N) and open reading frame 1ab (*ORF1ab*) genes, respectively (Figure 1C). Combining with 6 L/min of oxygen absorption by mask and 98% of pulse oxygen, the patient was diagnosed as COVID-19 state and was subsequently treated with antiviral drugs including interferon atomization, lopinavir, abidol, ribavirin, and anti-inflammatory medications like methylprednisolone sodiumsuccinate. Surprisingly, the patient had no travel history and no close contact with confirmed infected people, and none of her family members had been diagnosed and infected, making the source of infection unclear. The negative RT-qPCR tests also showed on February 13 and 14 (Figures 1D,E).

The chest CT scan on February 14 indicated partial absorption of pneumonia lesions than before (Figures 2B,E). With an oxygenation index of >300 mmHg, she was classified in moderate COVID-19 infection state on February 15.

From February 18, the patient symptoms improved and she maintained a normal body temperature, and the viral RNA detection remained negative (Figure 1F). The obvious absorption and improvements were observed on the CT imaging on February 19 (Figures 2C,F). Later on February 21, the quarantine of the patient was over and she was discharged.

## Serological Antibody Detection

The purified SARS-CoV-2 nucleocapsid protein (NP) and receptor-binding domain (RBD) of spike protein were coated to magnetic particles, and then a second antibody, conjugated with acridinium (which can react with substrates to generate a strong chemiluminescence), that binds with IgA, IgM, or IgG was added. We attempted to analyze specific IgA, IgM, and IgG antibodies in this patient's serum to confirm SARS-CoV-2 infection. The detected chemiluminescent signal over background signal was calculated as relative light units (RLU), which was measured using a fully automated chemical luminescent immunoanalyzer, Kaeser 1000 (Kangrun Biotech, Guangzhou, China). Meanwhile, our assays for IgA, IgM, and IgG specific to a virus surface antigen showed sensitivity of 98.6, 96.8, and 96.8%, and specificity of 98.1, 92.3, and 99.8%, respectively (4).

**Figure 3:**
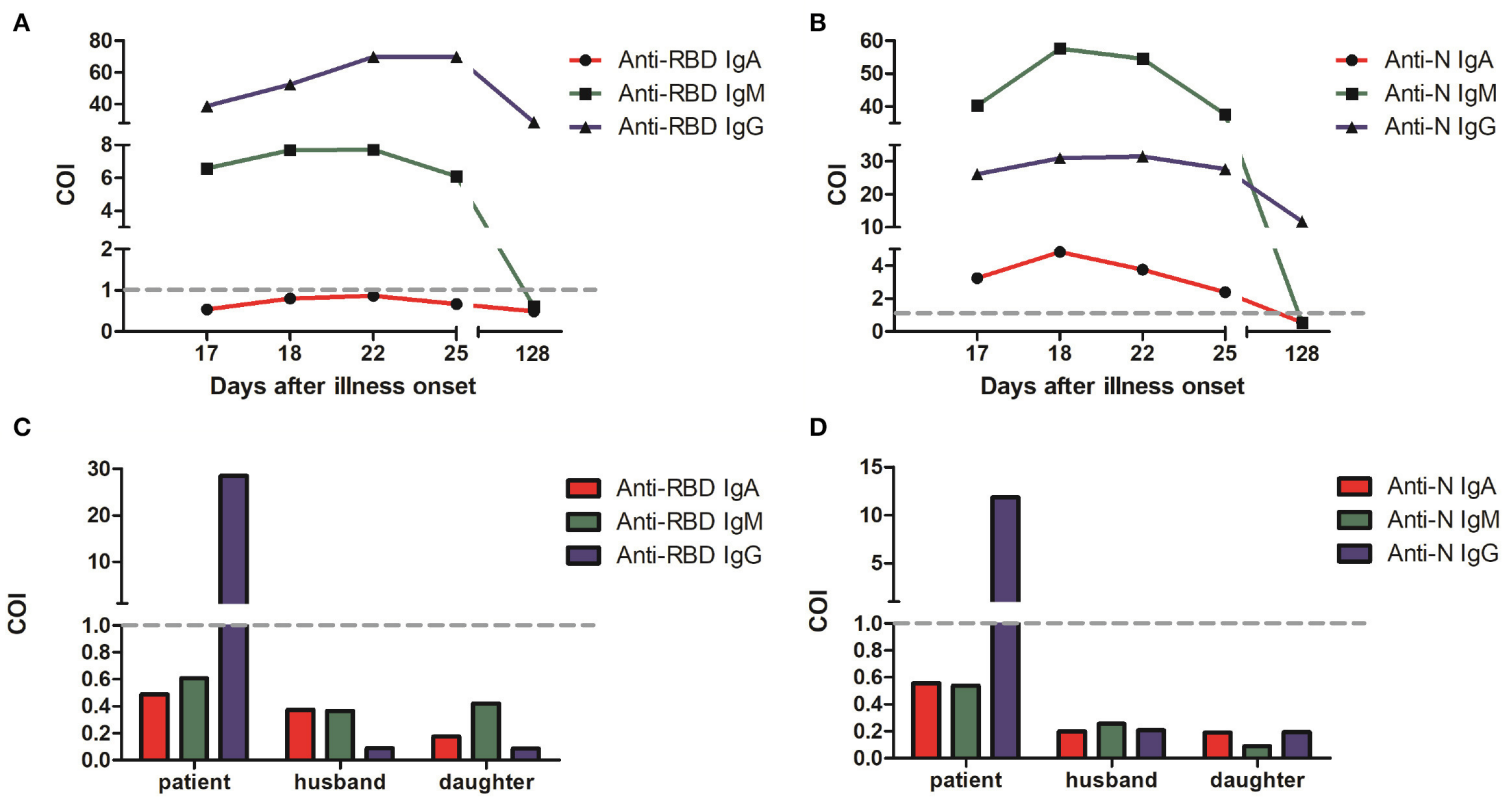
Anti-SARS-CoV-2 specific IgA, IgM and IgG levels in COVID-19 patient serum from the 17th to the 128th day after onset of illness. **(A)** Spike RBD-specific antibodies. **(B)** Nucleocapsid-specific antibodies. The values of RLU *(relative light units)* converted COI (Cut-Off Index) were indicated for three antibodies with red, green, and blue, respectively. Serological test results of the patient family members on June 2 in a follow-up visit with spike RBD-specific antibodies **(C)** and nucleocapsid-specific antibodies **(D)**. COI > 1 indicates positive results, and COI < 1 indicates negative results.

Serological testing of five antibodies showed positive results (Cut-Off Index or COI > 1), which confirming SARS-CoV-2 infection (Figures 3A,B). The median concentration of IgA and IgM reached the highest from the 18th to the 22nd day after the onset of illness, and then gradually declined. The median concentration of anti-RBD IgG was consistently increasing and remained high after 22 days from the onset of illness, which indicates the production of protective antibodies assisting in a patient recovery. However, the result of anti-RBD IgA was negative in this case. It was reported that COVID-19 severity is positively correlated with anti-RBD IgA antibody concentration (4). In this case, the patient was diagnosed as moderate COVID-19, which is consistent with that reported by Ma et al. (4).

The virus etiology of epidemiology in this patient was unknown or suspected to be probably infected through her work at her barbershop. This also may indicate that she acquired the virus from the community in her village provided that the virus has been distributed in the village before start of public health intervention. Further, it may be due to the presence of asymptomatic cases in the community. In a follow-up visit, we also conducted serological test with close contacts of this patient. The negative antibody test results showed that none of her family members including her husband and daughter were infected by SARS-CoV-2 (Figures 3C,D). SARS-CoV-2 RNA tests from throat swab samples in her family members were also negative.

## Discussion

By following our routine molecular diagnostic protocol, a total of six SARS-CoV-2 RNA RT-qPCR tests have been performed during the entire course of illness, and it took 17 days from onset of illness to finally diagnose the patient with COVID-19 primarily by clinical symptom in combination with CT.

The results of SARS-CoV-2 RNA tests depend on the viral load of the samples. SARS-CoV-2 RNA tests from swab samples could have been false-negative probably due to poor handling of samples during collection, preservation and transportation (5). However, in our hospital, we successfully diagnosed ~ 50 patients with RNA tests, among them no other COVID-19 patients had continuously false-negative results between 1 and 3 weeks after onset of illness during hospitalization before recovering, during which the virus is detectable in mixed samples of nasopharyngeal swabs and sputum. As a result, the continuously negative RNA test results of this patient are not likely due to technical issues.

Routes of infection and virus distribution might influence the RT-qPCR test accuracy. Recent studies have shown that the viral load in sputum was higher than that in the throat swabs (6). The weak positive RT-qPCR test results observed in our study also presented low viral load in this patient although deep sputum sample tested. Therefore, we speculated from this case that the viral load carried by the patient was too low, which resulted in several negative RT-qPCR test results during the early stage of the illness. Moreover, the absence of the virus in her close contacts could also be explained by the low viral load.

Chest CT is often as an immediate reference to screen highly suspected cases and evaluate the progression of COVID-19. However, it is difficult to clinically differentiate a SARS-CoV-2 infection through routine laboratory tests from other infections. Moreover, it is impractical to cover lung CT scans to all suspected patients in early diagnosis due to a shortage of medical resources. In the early stage of this patient with mild pneumonia often lack typical evidence to make a definitive diagnosis, and CT could be utilized to evaluate the progression of pneumonia and later to decide on discharge.

For asymptomatic patients with contact history, as well as symptomatic patients with negative RT-PCR results, specific antibody detection in the different stages of SARS-CoV-2 infection is essential for COVID-19 diagnosis (4, 7). IgA and IgM should be recommended in the early stage of COVID-19 diagnosis, and IgG should be recommended in the early to middle stages of the disease. Due to the non-specific characters of IgM (8), we highly recommend specific IgA/IgG or IgA/IgM/IgG combined tests to provide a more accurate diagnosis of COVID-19. Interestingly, we found the level of protective anti-RBD IgG remained high after patient recovery, which indicates that the patient has acquired anti-SARS-CoV-2 immunity.

Here it can be noted that negative RT-qPCR tests during the early stage of SARS-CoV-2 infection do not guarantee the absence of infection. Although it needs further studies, our case revealed that patients with low viral load might not transmit the virus to others through the common routes of infection as evidenced by the absence of infection in the family members. Based on that, this case provides a milestone for policymakers to revise policies regarding diagnostic modalities and the clinical decisions of rare cases.

## Data Availability Statement

All datasets presented in this study are included in the article/ supplementary material.

## Ethics Statement

The studies involving human participants were reviewed and approved by the Ethics Committee of the First Affiliated Hospital of Anhui Medical University (AF/SC-08/02.0). The patients/participants provided their written informed consent to participate in this study. Written informed consent was obtained from the individual(s) for the publication of any potentially identifiable images or data included in this article.

## Author Contributions

TJ and YX provide funding, designed the study, participated in data analysis, and wrote the manuscript. YL, XH, YT, TW, BW, HM, AK, WZ, and DZ designed the study, performed the majority of experiments, analyzed the data, and drafted the manuscript. HM, AK, and MZ participated in the revising of the manuscript. All authors contributed to the article and approved the submitted version.

## Conflict of Interest

The authors declare that the research was conducted in the absence of any commercial or financial relationships that could be construed as a potential conflict of interest.
